# Case study: Gait assessment of a patient with hallux rigidus before and after plantar modification

**DOI:** 10.1016/j.ijscr.2023.109197

**Published:** 2023-12-24

**Authors:** Roberto Tedeschi

**Affiliations:** Department of Biomedical and Neuromotor Sciences (DIBINEM), University of Bologna, Bologna, Italy

**Keywords:** Case report, Baropodometric examination, Hallux limitus, Insole, Motion sensors

## Abstract

**Introduction and importance:**

Hallux rigidus (HR) is a degenerative arthritis affecting the first metatarsophalangeal joint (MTP), leading to pain and functional impairment, particularly during the propulsive phase of walking. The prevalence of HR is about 2.5 % in individuals over 50, but younger individuals can also be affected, as demonstrated in this case.

**Case presentation:**

We report the case of a 26-year-old patient with a body mass index (BMI) of 20.2, who has been suffering from HR for 5 years. The patient presented with walking difficulties, characterized by a limp and impaired propulsion phase, and pain in the right foot due to HR. A comprehensive gait assessment was conducted using a baropodometric platform and integrated smartphone motion sensors. Following the diagnosis, a non-surgical intervention involving the application of a compressed cotton felt foot orthosis at the MTP plantar area was initiated. This intervention aimed to alleviate pain and improve the functional mobility of the right big toe. Post-treatment assessments showed an increase in the big toe's mobility from 0 degrees to 35 degrees, as measured by a digital goniometer.

**Clinical discussion:**

The application of a soft support, such as compressed cotton felt, at the plantar area of MTP, demonstrated a potential non-surgical therapeutic approach to improve gait and reduce discomfort in HR patients.

**Conclusion:**

This case study underscores the potential benefits of plantar modification in the management of HR.

## Highlights

• Incidence of hallux rigidus (HR) observed in an unusually young patient, diverging from typical age-related onset patterns.
• Detailed case report of a 26-year-old woman experiencing significant right foot pain attributed to early-onset HR.
• Innovative use of a baropodometric platform combined with smartphone technology for comprehensive foot function assessment.
• Successful reduction of pain through sustained application of tailored plantar support, demonstrating a non-surgical intervention's potential efficacy.

## 1 Introduction

Hallux limitus and its more severe form, hallux rigidus (HR), result from osteoarthritic degeneration of the first metatarsophalangeal joint (MTP), leading to swelling, stiffness, pain, and functional limitation [1]. The prevalence of HR is about 2.5 % in individuals over 50, but younger individuals can also be affected, as demonstrated in this case [2], and is the primary cause of foot arthritis, with an increasing incidence in the elderly population [3]. Hallux limitus and hallux rigidus are common foot conditions that can be highly debilitating. To assess the utility of a modification to reduce symptoms or improve foot function, some healthcare professionals, such as podiatrists, occasionally use a piece of compressed cotton felt as a test. It's worth noting that, to our knowledge, there are no examples of using cotton felt in this manner in the medical literature. Radiographic signs such as joint space narrowing and subchondral sclerosis have been observed in about 10 % of patients aged 20 to 34, suggesting that this condition can begin as early as adulthood [4]. Progression to hallux rigidus is associated with significant to complete loss of joint function, chronic pain, and a decrease in quality of life [5,6].

In this study, we present the case of a young woman suffering from persistent pain in the first metatarsophalangeal joint (MTP) of the right foot, which had been felt over the past five years, and who was eventually diagnosed with initial osteoarthritis of the MTP, following a consultation at the Orthopedic Institute. We also reviewed similar cases previously reported in the literature [7]. This case highlights the importance of recognizing that, even at a young age, initial osteoarthritis of the MTP may only present as nonspecific foot pain, before focusing on the affected area. Due to its rarity at such a young age and the anatomical complexity of the joint, this arthritic condition presents diagnostic, functional, and rehabilitation challenges [8].

Besides osteoarthritis, HR and HL can stem from various factors, including an elevated or abnormally long metatarsal joint or first metatarsal bone, injuries like toe sprains, a fractured toe, or significant toe stubbing, anatomical variations, and developmental impairments, as discussed in a case report. Overuse from activities that continually stress the toes is also a contributing factor [9].

Compressed cotton felt is available in various thicknesses, with the most commonly used ranging from 2 mm to 5 mm. In this study, we used compressed cotton felt with a thickness of 5 mm. The aim of this study was to evaluate the effectiveness of adjusting a foot orthosis using compressed cotton felt. We sought to determine if this simple and cost-effective method could improve functionality and alleviate pain in patients suffering from hallux rigidus, without having to wait for the fabrication of a custom orthosis**(**Table 1**).** This research is especially relevant given the lack of information in the literature on this topic.

**Table 1:** Spatio-temporal parameters of walking measured with smartphone.

Parameter	J-2 (October 20, 2022)	J-1 (October 21, 2022)	J0 (Visit - October 22, 2022)	J + 1 (October 23, 2022)	J + 2 (October 24, 2022)
Speed (m/s)	5.10 SD: 1.54 SE: 0.68	4.75 SD: 1.98 SE: 0.88	5.45 SD: 1.27 SE: 0.57	5.50 SD: 1.47 SE: 0.65	5.00 SD: 1.36 SE: 0.60
Stride Length (cm)	71.00 SD: 2.50 SE: 1.12	61.00 SD: 2.00 SE: 0.89	72.00 SD: 2.60 SE: 1.16	77.50 SD: 2.80 SE: 1.25	75.00 SD: 2.70 SE: 1.21
Double Support (sec)	28.95 SD: 1.45 SE: 0.65	26.30 SD: 1.31 SE: 0.59	27.25 SD: 1.36 SE: 0.61	29.25 SD: 1.46 SE: 0.65	28.06 SD: 1.40 SE: 0.63
Walking Stability	Appropriate	Inappropriate	Appropriate	Appropriate	Appropriate

Note: The days are indicated in relation to the visit (J0) when the cotton support was applied. J-2 and J-1 represent the days before the visit without the cotton support, while J + 1 and J + 2 represent the days after the visit with the cotton support, SD: Standard Deviation, SE: Standard Error.

### 1.1 Patient information

**Fig. 1:**
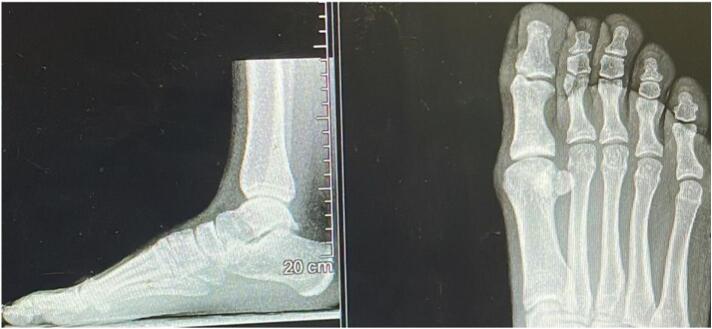
X-rays foot.

**Fig. 2:**
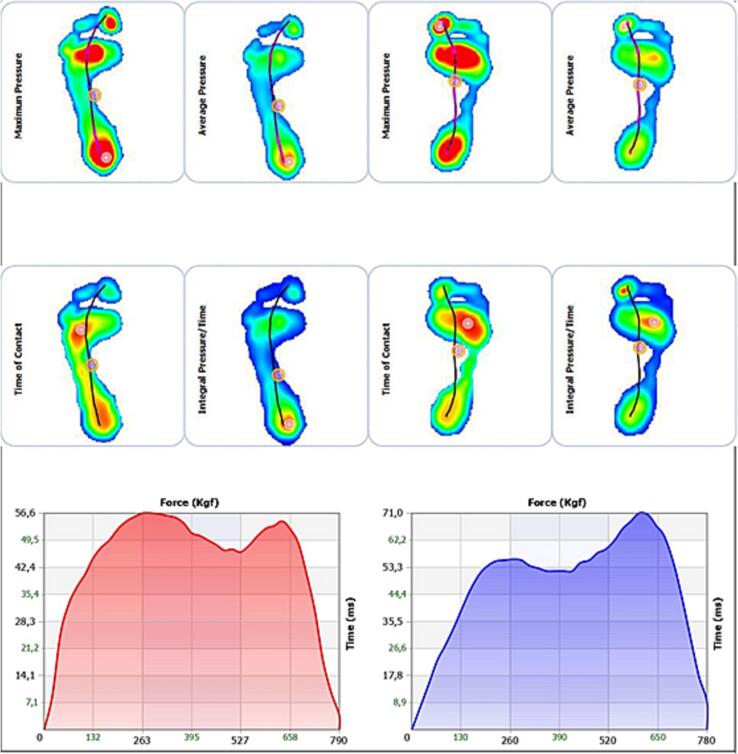
Dynamic Baropodometric Platform: plantar pressures during walking [22].

**Fig. 3:**
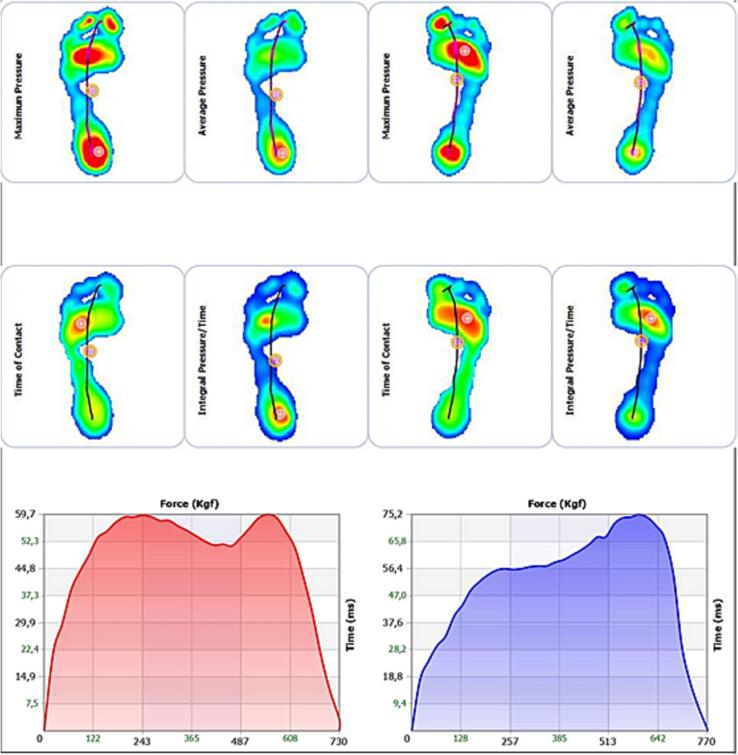
Dynamic Baropodometric Platform: plantar pressures during walking with support of the 1st MTP of the right foot [22].

A 26-year-old female, with a body mass index of 20.2, presented to the Orthopedic Institute with pain in the metatarsophalangeal joint (MTP1) of the right foot, rated at 6/10 on the NPRS (Numeric Pain Rating Scale) for over 5 years. The pain did not occur at night but increased after her activity as a Latin American dancer, and she couldn't find any physical posture that would alleviate the pain. She had taken anti-inflammatories for a short period, but they did not alleviate the pain. She had no history of fever, trauma, or systemic disease. She had previously undergone corticosteroid infiltration a year earlier, which had been beneficial for a few months, and an X-ray (Fig. 1) had confirmed the initial degeneration of the MTP joint, but she had never tried manual foot physiotherapy or orthopedic insoles to alleviate the pain. The clinicians chose X-rays for the initial diagnosis of hallux rigidus based on their effectiveness in detecting bony alterations and joint changes, offering a less invasive and more accessible approach compared to CT scans. Although CT scans provide more detailed images, X-rays are often sufficient for an accurate diagnosis of hallux rigidus and are preferred for their practicality, lower cost, and reduced radiation exposure. [[10], [11], [12], [13], [14]]. On objective examination, the active movement of the MTP joint of the big toe showed modest limitation in the sagittal plane, measured using an analog goniometer (10–15 degrees), but an increased sensation of pain throughout the foot, measured using the NRS scale (6–7). The Jack test was performed to passively test the capsular tissue of the MTP joint, and the Tip Toe Standing test to assess load-bearing capacity, both of which turned out positive. The “Jack test” is a passive stress test used to assess the stability of the capsular tissue of the MTP joint. The “Tip Toe Standing Test” is used to evaluate load-bearing capacity and involves standing on the ball of the foot to see if it causes pain or instability in the joint. Often these two tests are performed one after the other; when one or both are positive, there is a more or less marked difficulty in dorsalizing the first ray, the propulsive phase of walking is affected by compensatory changes in gait. Unfortunately, the validity of these tests has not been calculated. The Jack test [15] has an intraclass correlation coefficient (ICC) of 0.997 (0.995–0.999) [8]. On palpation, a palpable mass was found in the dorsal region of the MTP joint of the right foot, which, according to imaging, appeared to be a small osteophyte. When walking barefoot, a clinically apparent evasive limp with avoidance of the propulsive phase was observed. The patient was given a soft compressed cotton (felt) support to aid the MTP joint, aiming to make walking more functional and alleviate pain. This type of support is commonly used in podiatry to temporarily relieve foot overload areas but is seldom used as a trial to evaluate a future orthopedic orthosis. The literature is lacking in this area, and this article is the first to evaluate the use of felt as evidence in this manner. She was assessed using a baropodometric platform (Fig. 2, Fig. 3), with and without the soft support, to evaluate the foot's pressure parameters. Moreover, thanks to the ‘Health’ application integrated into Apple smartphones, we were able to automatically monitor the spatio-temporal walking parameters, such as speed, step length, and double support, in the days before and after administering the support.

### 1.2 Clinical findings

**Fig. 4:**
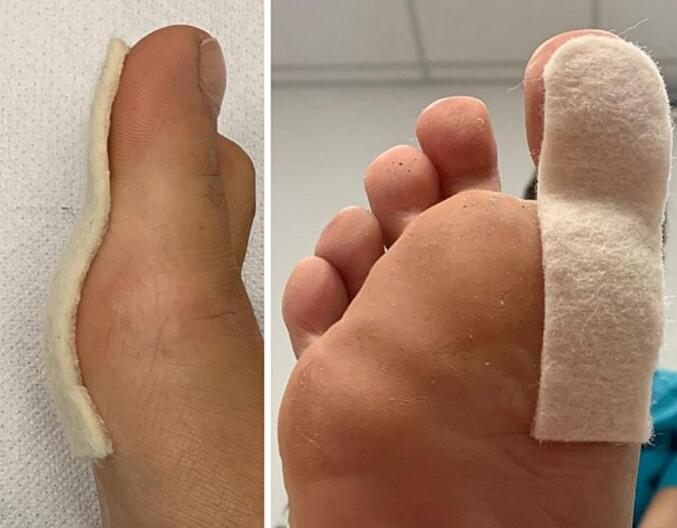
Plantar Support Application on Hallux.

Upon further diagnostic evaluation, radiographic imaging was also conducted to assess the structural integrity of the MTP joint. The X-rays (Fig. 1**)** revealed initial signs of joint degeneration, corroborating the clinical findings and adding another layer of diagnostic certainty. No significant bone deformities or osteophytes were observed, which ruled out the need for immediate surgical intervention. The radiographic findings, in conjunction with the clinical tests and patient-reported outcomes, provided a comprehensive understanding of the condition, further validating the effectiveness of the soft cotton support **(**Fig. 4**)** in alleviating symptoms and improving joint function. This case study adheres to the SCARE [16](Surgical Case Report) guidelines for reporting surgical case studies.

### 1.3 Timeline

Day 0: Initial presentation of symptoms (Patient reports pain and stiffness in the right foot MTP joint).

Day 3: Application of plantar support (Custom plantar support is applied after assessment) (Fig. 4).

Day 5: Assessment of initial response (Initial reduction in pain and improvement in functionality observed, cotton felt removal).

Day 10: Follow-up via telephone (Patient reports sustained pain relief and comfort from the plantar support).

### 1.4 Diagnostic assessment

Jack's Test [15],Tip Toe Standing test [17], Baropodometric Platform [18], Smartphone [19].

The Jack's Test helps evaluate passive dorsiflexion of the joint, while the Tip Toe Standing Test provides insights into the patient's ability to bear weight on the affected joint. Both contribute to a more comprehensive and informed diagnosis.

### 1.5 Therapeutic intervention

The chosen intervention was a non-surgical method involving the application of compressed cotton support, selected for its conservative nature suitable for the young patient's condition of hallux rigidus. The intervention entailed applying the cotton support to the plantar area of the foot in a clinical setting. The application was carried out by an experienced podiatrist, specializing in non-surgical foot treatments.

Recent advancements in computational simulation have shown promising potential in the field of medical investigation, particularly in orthopedics. demonstrate Studies [20,21] the efficacy of computational models in assessing stress and wear in prosthetic joints under various conditions.

1. Speed (m/s):Before Application (J-2, J-1): The speed was lower before the visit, averaging 4.925 m/s.

On the Day of Visit (J0): There's an increase in speed to 5.45 m/s.

After Application (J + 1, J + 2): Post-application, the speed slightly decreased on J + 2 compared to J + 1, but both values are higher than the pre-application days.

2. Stride Length (cm): Before Application: The stride length was shorter before the application, with a noticeable decrease on J-1 (61.00 cm).

On the Day of Visit: It increased to 72.00 cm.

After Application: Post-application, the stride length increased further, peaking at 77.50 cm on J + 1.

3. Double Support (*sec*): Before Application: The time spent in double support was lower before the application.

On the Day of Visit: There was a slight increase to 27.25 s.

After Application: The time increased further on J + 1 and then slightly decreased on J + 2 but remained higher than pre-application values.

4. Walking Stability: Before and After Application: Walking stability was noted as “Appropriate” on all days except for J-1, where it was “Inappropriate.”

The data reflects changes in walking parameters following the application of compressed cotton support to the MTP joint. Notably, there was an improvement in both walking speed and stride length after the support application. Speed increased from an average of 4.925 m/s before the visit to 5.45 m/s on the day of the visit and remained higher post-application. Similarly, stride length showed an increase, reaching its peak the day after the application. The time spent in double support also increased post-application, indicating a possibly more cautious gait. Walking stability was generally maintained as ‘Appropriate’ throughout, with an exception on the day before the visit. These observations suggest that the compressed cotton support might enhance mobility and comfort for individuals with MTP1 joint issues.

## 2 Follow-up and outcomes

After the application of the compressed cotton support under the head of the 1st metatarsal of the right foot, we reviewed her during the follow-up about a week later. Her pain was 1/10 on the NPRS scale, and she was prescribed orthotics with the same modification as the applied support. A follow-up examination has not yet been conducted.

## 3 Discussion

Hallux rigidus (HR), characterized by stiffness and pain in the big toe joint, represents a significant challenge in foot pathology. This condition arises from wear and tear on the joint over time or due to repetitive trauma or acute injury [23,24]. The symptoms, predominantly pain and limited mobility, necessitate an early and accurate diagnosis to prevent further degeneration and the need for surgical intervention [25].

Our study's findings regarding the increase in plantar pressures from 120.4 kPa to 125.8 kPa post-treatment align with [26]. This improvement indicates enhanced support in the forefoot, crucial for the effective management of HR. Despite the limitations of a case report format, our study provides valuable insight into symptom assessment and the objective evaluation of joint stability, using methods like the NPRS scale and a baropodometric platform [27].

The multidisciplinary approach in diagnosing and treating the patient in our study, especially considering the patient's young age, emphasizes the complexity and necessity of a comprehensive evaluation in HR cases [28]. The use of smartphone technology for patient follow-up, particularly in tracking spatiotemporal parameters, is an innovative aspect of this study. It highlights the potential of modern technology in enhancing patient care [29].

Interestingly, the application of soft compressed cotton support in the plantar area of the MTP joint suggests a promising non-surgical intervention. This approach not only improved foot function but also limited compensations, raising questions about its mechanism of action on the accessory joint movements [30]. Our findings, though preliminary, suggest that such interventions could lay the groundwork for accurate plantar orthosis prescriptions [31].

The slight deterioration in patient outcomes on the *sec*ond day post-intervention, potentially due to the material properties of the felt, underlines the need for further research into the durability and long-term efficacy of such treatments.

In conclusion, our study adds to the existing body of knowledge by using compressed felt as a therapeutic and evaluative tool in HR, demonstrating the potential of non-surgical approaches. However, more high-quality studies are necessary to confirm these findings and explore their generalizability.

The integration of imaging diagnostics, as seen in our case where radiographic imaging confirmed the initial degeneration of the MTP joint, plays a crucial role in treatment planning and long-term management of HR. This comprehensive diagnostic approach is essential for optimizing patient outcomes in conditions as potentially debilitating as hallux rigidus.

### 3.1 Evaluation tool validity in our study

• Baropodometric Platform: This tool is established for assessing foot biomechanics, though its effectiveness depends on precise calibration and expert interpretation.
• Smartphone Application: The app's innovative approach offers convenience, but its validity is contingent on sensor accuracy, which may not match the precision of traditional clinical tools.

While the plantar support showed positive patient feedback, its effectiveness needs further validation through controlled trials and comparison with other treatments. The tools used show promise but require more extensive validation in future research.

### 3.2 Weaknesses of the case report: limited follow-up

One of the primary weaknesses of our case report is the insufficient length of follow-up. While the initial outcomes post-intervention showed promising results, the short-term nature of our observation period limits our ability to assess the long-term efficacy and sustainability of the treatment. A longer follow-up period would provide valuable insights into the durability of the therapeutic effects, potential long-term complications, and the need for ongoing interventions.

### 3.3 Patient perspective

An important aspect of our case report is the patient's own perspective on the treatment received. In this instance, the patient expressed significant satisfaction with the non-surgical intervention. They reported a notable reduction in pain and an improvement in foot functionality, which positively impacted their daily activities and overall quality of life. This patient feedback underscores the effectiveness of the treatment from the viewpoint of those directly experiencing its benefits, adding a crucial dimension to the evaluation of the therapeutic intervention.

## Ethical approval

Ethical approval is not a requirement at our institution for reporting individual cases or case series.

## Funding

There is no source of funding.

## CRediT authorship contribution statement

RT contributed to conception and design of the study; RT to data acquisition, RT to data analysis and interpretation; RT contributed to draft the manuscript; RT contributed to the critical revision for important intellectual content. All authors read and approved the final version of the manuscript.

## Guarantor

Roberto Tedeschi.

## Informed consent

The subject signed a standardized informed consent. Ethics committee: not required.

## Consent

Written informed consent was obtained from the patient for publication of this case report and accompanying images. A copy of the written consent is available for review by the Editor-in-Chief of this journal on request.

## Declaration of competing interest

As the sole author of this manuscript, I declare that there is no financial or personal relationship with any individual or organization that could inappropriately influence or bias the work presented in this paper. There has been no external funding for this research, and as such, there are no financial conflicts of interest to report.
